# Reining in Angola’s yellow fever outbreak

**DOI:** 10.2471/BLT.16.031016

**Published:** 2016-10-01

## Abstract

Rosa Moreira tells Andréia Azevedo Soares how Angola reined in its worst yellow fever epidemic in 30 years.

Q: Since the first cases of yellow fever appeared earlier this year, 884 confirmed cases have been reported including 121 deaths. How did the outbreak start?

A: In the beginning, we didn't know what we were dealing with. The first patients were Eritreans living in Viana district in the capital, Luanda. They had high fever and haemorrhage. When the fifth patient died, the clinic where they were receiving treatment reported these cases to the health ministry. When we arrived at the clinic to investigate, one of the patients was still alive. We took samples and did tests. We looked for people he had been in touch with. The survivor and two of his contacts tested positive for yellow fever. A week later, on 19 January, our results were confirmed by the National Institute for Communicable Diseases in South Africa. Now we have laboratory capacity to do these tests ourselves. It turned out that the first patients had fake yellow fever vaccination certificates, which delayed a correct diagnosis.

“In the beginning, we didn't know what we were dealing with.”

Q: How did you mount the response?

A: The government set up a task force to lead the response and launched a five-part plan. The first part was active surveillance. We reinforced laboratory capacity to allow for early detection and notification of new cases. The second part was case management. With the help of Médecins Sans Frontières, we developed case management guidelines and distributed these to different provinces because there is no specific treatment for yellow fever. We also provided health workers with a flowchart indicating six health facilities where people with severe disease should be referred. By February, about 30% of some 300 people with confirmed yellow fever had died. After implementing our plan, case–fatality dropped to 11%. The third part was mass vaccination, and the fourth was integrated vector control measures to lower the density of the *Aedes aegypti *mosquito that carries yellow fever, as well as dengue, chikungunya and Zika viruses. The fifth part of the strategy was risk communication and social mobilization.

Q: How did you start?

A: We began working in Viana, a marketplace attracting people from all over Angola. It’s difficult to control the movement of people and the area has major sanitation problems. Mosquito density was high, so we carried out a mass distribution of larvicide and a social mobilization campaign to explain to people how to use it. We also did indoor and outdoor spraying with insecticides. Initially we faced resistance: some people kept the larvicide at home and did not use it. So we asked community leaders to help us persuade people to join the campaign. As a result of these vector control measures, the mosquito density level fell substantially.

Q: The Emergency Committee under the International Health Regulations advised WHO on 31 August that the outbreak did not constitute a public health event of international concern. How did you succeed in bringing the outbreak under control?

A: We have been conducting fixed post and mobile vaccination campaigns in 73 districts. By 7 September about 16 million individuals, 65% of the Angolan population, had been vaccinated – most of them in reactive campaigns, where local transmission was confirmed. In addition, 3 million of these individuals were vaccinated in a preventive campaign in August and September, and a further 2 million will be vaccinated this month. Supplementary vaccination activities have also been going on in the three districts where vaccination coverage has not reached 80%. 

*Q: The WHO Strategic Advisory Group of Experts (SAGE) on Immunization recently advised that there was evidence that one fifth of a standard vaccine dose protects an individual against the disease for at least 12 months and could be used in an emergency if there are vaccine shortages. In the Democratic Republic of the Congo reduced doses of vaccine have been used in Kinshasa because 18 million doses were needed in July and only 11 million were available globally. Have you vaccinated people with reduced doses in Angola?*

A: No. Our aim is to vaccinate the whole population to ensure lifelong immunity. We hope that in so doing, in addition to routine vaccination for children (covering above 80%), we won't have to face another yellow fever epidemic like the current one. This decision also takes account of the fact that vector populations are present in all provinces of our country.

Q: Most yellow fever cases have been in and around Luanda, where the immunization campaign started in February. What were the challenges for these campaigns?

A: At first the challenge was planning and managing mass vaccination in a short space of time. After that we had a different challenge. When you don’t vaccinate people quickly, new problems can occur. Some people refuse the jab because “a relative had died after being vaccinated”. We tried to explain that the person may have died because he or she was already infected. It is often difficult to convince them. We have also been facing resistance to vaccination from people based on their cultural and religious beliefs. So there are several reasons why it’s difficult to achieve full coverage.

Q: What was different about this yellow fever outbreak?

A: Population density has been the big challenge. The last yellow fever outbreak in our country was in 1988, with 37 cases and 14 deaths. Since then, our population has more than doubled from about 12 million to more than 25 million people. In 1988, the population of Luanda province was less than 2 million. Today it is almost 7 million. Today’s population is highly mobile. In 1988, we were in the midst of civil war, most people did not travel and we were able to immunize the whole population of Luanda province. This time, we could not do that initially because there was not enough vaccine for all 7 million people. The vaccine is produced by only four manufacturers based in Brazil, France, the Russian Federation and Senegal, and we have been receiving vaccine supplies in batches. Angola had the funds to pay the full cost of the vaccine, but we still could not get it as quickly as we wanted because of limited global supplies.

Q: How did you overcome the problem of limited supplies?

A: By 7 September we had received 18.1 million doses from the International Coordinating Group (ICG) for the emergency global stockpile of yellow fever vaccines – as well as additional supplies from Bio-Manguinhos in Brazil. The ICG provided vaccine supplies for reactive campaigns in Luanda. The vaccine for other provinces came from stocks reserved for routine immunization or preventive campaigns in other African countries. 

Q: What has Angola done to rein in the international spread of yellow fever?

A: Some time ago unvaccinated people travelled to Angola, got yellow fever and left the country with the disease. From February to the end of May, we vaccinated more than 530 incoming travellers at Quatro de Fevereiro International Airport in Luanda. For example, more than 100 people from Portugal were vaccinated on arrival, but the problem with such measures is that people are only protected from 10 days after vaccination. In the meantime, they can become infected. So it’s a big window. In light of the International Health Regulations, the Angolan health ministry issued a directive regulating the control of yellow fever vaccination when people enter and exit the country and for some domestic travel. We control their exit at checkpoints and no one can leave without a yellow fever vaccination certificate. But we have more than 5000 kilometres of borders, most of which are not strictly controlled. We have also prioritized vaccination of people living in border areas. Angola’s public health authorities are also taking measures to prevent the sale of fake yellow fever vaccination certificates within the country, but it’s difficult to control the entry of foreigners with fake certificates when the certificates have correct lot numbers.

“Hopefully there will be no more cases and we can safely say that the outbreak is over.”

Q: Is the outbreak a sign of changing trends in vector-borne diseases in southern Africa?

A: It’s difficult to say. The outbreak coincided with unusually heavy rains and a severe El Niño weather pattern. We are also suffering from an economic crisis and poor sanitary conditions. All these factors created a fertile environment for an increase in the mosquito population. The outbreak reached its peak in February and has been declining since. We have much more vaccine now than we had earlier in the epidemic. The response interventions are involving communities successfully. The dry season arrived in May and since then the mosquito population has diminished. I am optimistic: we had two confirmed yellow fever cases last month but, before that, there had not been any cases since June. Hopefully, soon there will be no more cases and we can safely say that the outbreak is over.

Q: What drew you to the field of epidemiology?

A: It was my childhood dream to become a doctor. Before I could study medicine, I was sent to the Nursing School in Angola. I learned a lot and when I became a doctor, I realized that there were more questions than answers. That’s how I discovered that I wanted to do research and study epidemiology. Doctors help individual patients, but public health can provide answers to help communities. That’s why I decided to study public health in Zimbabwe. After that, I moved back to Angola to train students in field epidemiology. It was the right decision because we need field epidemiologists now more than ever.

Q: Why does Africa need epidemiologists now more than ever?

A: We are disease detectives. In Angola we are on the frontline of the national response to the yellow fever outbreak. We are trained to look for clues. We run tests; we ask, “Who is sick? When and where did they get sick? “What are their symptoms?” and then we look at the data and try to solve the puzzle. We once had a malaria outbreak in eastern Angola and I saw so many children struggling to survive. I was deeply moved because they were malnourished and I realized that not only malaria was killing them. This experience made me want to work even more on research. If we want to detect outbreaks early on, we need more field epidemiologists. We are the ones who can help countries understand what is happening and how to set up surveillance systems and response interventions.

